# A case of Lymphoplasmacyte-rich meningioma mimicking pachymeningitis

**DOI:** 10.1186/s12883-022-02794-z

**Published:** 2022-07-29

**Authors:** Yue Zhang, Xiang Zhang, Abhijeet Kumar Bhekharee, Zunguo Du, Shuguang Chu

**Affiliations:** 1 Department of Neurology, Huashan Hospital, Fudan University, Shanghai, China; 2 Shanghai Medical College, Fudan University, Shanghai, China; 3 Department of Pathology, Huashan Hospital, Fudan University, Shanghai, China; 4 Department of Radiology, Pudong New District, Shanghai East Hospital, Tongji University School of Medicine, No. 150 Jimo Road, Shanghai, 200120 China

**Keywords:** Lymphoplasmacyte-rich meningioma, Pachymeningitis, En plaque meningioma

## Abstract

**Background:**

Lymphoplasmacyte-rich meningioma (LPRM) is a rare form of meningioma characterized by prominent lymphoplasmacytic infiltrates into the tumor. Report of flat growth of LPRM mimicking pachymeningitis is rare in the literature.

**Case presentation:**

A 55-year-old female who suffered from episodes of headache and seizures has been diagnosed with pachymeningitis for 4 years because post contrast brain MRI demonstrated enhanced carpet-like dura lesion in the left frontal lobe. The lesion kept unchanged on yearly follow-ups until a recent brain MRI found the lesion grew significantly into a mass. The lesion was resected and pathology suggested LPRM.

**Conclusion:**

LPRM may present as carpet-like growth pattern on MRI. Long-term follow-up in patients with pachymeningitis is necessary.

## Background

Lymphoplasmacyte-rich meningioma (LPRM) is an extremely rare histological variant of meningioma characterized by prominent lymphoplasmacytic infiltrates into the tumor. It accounts for 0.51% of intracranial meningiomas [1]. Meningiomas usually manifest as single or multiple pachymeningeal masses on MRI while some rare variants, especially *en plaque*meningiomas (EPM) may present with carpet-like growth pattern. Occasionally such growth pattern can also be observed in LPRMs [1–8]. Herein, we describe a case of LPRM mimicking pachymeningitis on brain MRI initially. Four years after onset, the lesion grew into an obvious mass in the left frontal lobe. The diagnosis of LPRM was confirmed by dural biopsy.

## Case presentation

**Fig. 1:**
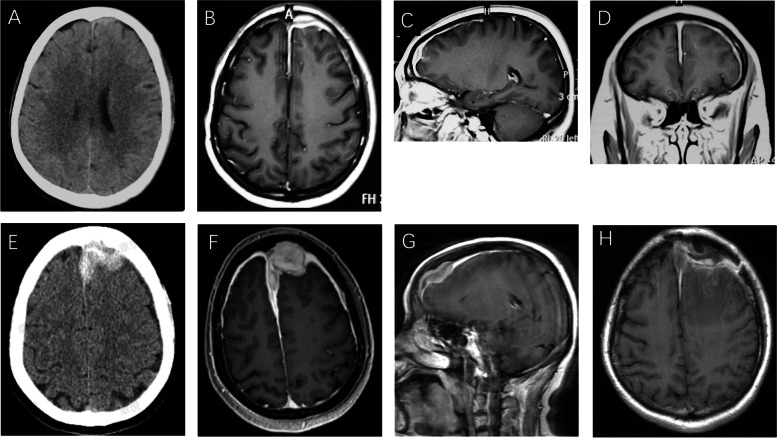
Neuroimages of LPRM. Brain CT in 2016 (**A**) showed left frontal dural hyperdensity. Axial (**B**), sagittal (**C**) and coronal (**D**) post contrast T1WI showed diffuse dural enhancement involving both convexity and falx cerebri. Brain CT in 2020 (**E**) showed left frontal dural hyperdense mass. Axial (**F**) and sagittal (**G**) post contrast T1WI showed significant enlargement of left frontal lesion and more diffuse dural enhancement than that in 2016. After corticotherapy MRI showed significant resolution of the dural enhancement. (**H**)

A 55-year-old female was admitted for a 4-year history of recurrent psychiatric problems, headache, and seizures. In June 2016, the patient developed irritation, auditory hallucination and delusion. Brain CT revealed hyperdensity in the left frontal dura matter (Fig. 1 A). Symptoms were soon controlled by risperidone. On 11 November 2016, she suffered from acute severe headache, drowsiness, dullness and seizures. Intravenous diazepam and valproate were administered to control seizures. Post-contrast MRI of brain revealed lineal dural enhancement in the left forehead and pachymeningitis was considered (Fig. 1 B-D). Differentials included meningioma, lymphoma, neurosarcoidosis, Rosai Dorfman disease and Erdheim Chester disease. However, the patient refused to receive dural matter biopsy. In the past 4 years, she had suffered from episodic psychiatric problems, headache and seizures. Intravenous mannitol and diazepam could alleviate symptoms and no long-term medications were given. Brain MRIs were performed every year and they showed the lesion unchanged until December 2019 when brain CT and MRI revealed that the lesion had grown into a mass (Fig. 1 E–G). Past history was unremarkable except chronic hepatitis B and 10-year-long well controlled hypertension.

**Fig. 2:**
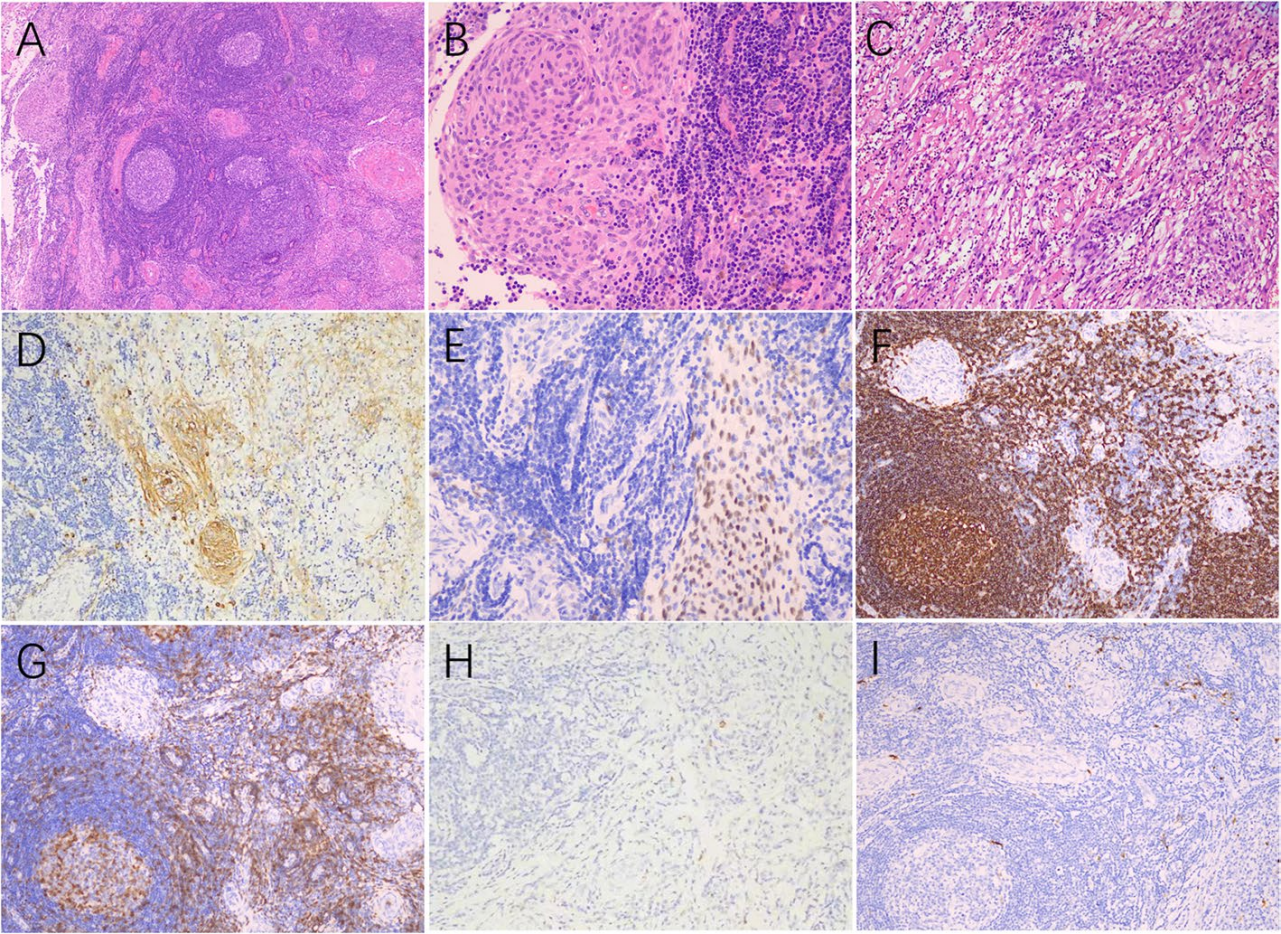
Histopathological changes of the lesion. The mass lesion showed scattered nests of meningothelial cells in a mixed inflammatory background with follicular center formation (**A**, HE*40). Nests of neoplastic meningothelial whorls were identified in lymphocytes and collagen (**B**, **C**, HE*100). Neoplastic cells were positive for EMA (**D**, *100) and PR (**E**, *100) in immunohistochemistry staining. Lymphocytes were composed of CD20 positive B cells (**F**, *100) and CD3 positive T cells (**G**, *100). Rare plasma cells were positive for IgG4 (**H**, *100). Fewer S100 positive cells existed (**I**, *100)

She was admitted in April 2020. On admission, she was alert, oriented but slightly apathetic. Cognitive function was not impaired. Chaddock sign was present in the right. Laboratory tests were unremarkable except elevated serum IgG to 18.1 g/l (reference range: 7.51–15.6). Lumbar puncture revealed an opening pressure of 200mmH_2_O. Analysis of cerebrospinal fluid (CSF) showed normal cell count, glucose, protein and chloride levels. CSF IgG4 was undetectable. Malignant cell was not found in CSF. The patient received left frontal dural mass biopsy, and it showed clustered nests of meningothelial cells in an abundant inflammatory and collagenated background (Fig. 2 A-C). These meningothelial cells were positive for VIM, EMA, PR, and SSTR2 (Fig. 2 D, E). The inflammatory cells were mixed and polyclonal, where lymphoma and histiocytosis were excluded. They were partially positive for CD20, CD3, CD79a, CD68, and CD138 (Fig. 2 F, G). Fewer IgG4 + plasma cells were found (Fig. 2 H). Staining of CD1a, kappa/lambda chains, and S100 was negative (Fig. 2 ). B cell gene rearrangement showed negative results. LPRM was thus considered. After surgery, the patient became lethargic. Intravenous methylprednisolone of 500 mg was administered daily for 3 days and then tapered off. Her mental status improved rapidly and dural enhancement on MRI fainted 2 weeks after corticotherapy (Fig. 1 H). Six months after surgery, the patient was asymptomatic and the MRI manifestation was similar to that of half a year ago.

## Discussion and conclusion

LPRM is an extremely rare histological variant of WHO grade I meningioma, characterized by infiltration of lymphocytes and plasma cells in the tumor. On MRI, most LPRMs present as dural masses, but flat growth has been occasionally described in the literature [1–8]. Several case reports mentioned they might mimic idiopathic hypertrophic pachymeningitis (IHP) [4–6] which is a disease characterized by inflammation and fibrosis of the dura mater without determined pathogenesis. In the present case, the initial brain MRI revealed lineal enhancement without obvious mass, so the diagnosis of IPH was preferred. Glucosteroids is the mainstay treatment for IPH. Long-term stability without corticotherapy is rare in this disease [9]. In this case, however, the episodic symptoms could resolve spontaneously which made the diagnosis of IPH doubtable. Other differential diagnosis included various neoplastic or non-neoplastic conditions. EPM is a special growth pattern of meningioma when tumor infiltrates the dura matter in a carpet-like appearance, rendering a thin layer in the dura. Whereas they are more inclined to involve sphenoid wing rather than convexity [10]. Dramatic osseous destruction is also an important feature. Primary pachymeningeal lymphoma accounts for 6.3% of primary CNS lymphomas [11]. Leptomeningeal involvement and parenchymal infiltration are common in pachymeningeal lymphoma. Non-neoplastic diseases, such as IgG4-related pachymeningitis, neurosarcoidosis, Rosai Dorfman disease, or Erdheim Chester disease was not supported by ancillary testing or pathology.

The nature of LPRM has not been fully understood. Some authors regard it as a mechanism of host immune resistance to the tumor [12]. So, LPRM may bear some features of inflammation, such as anemia, polyclonal gammopathy and peritumoral brain edema [2]. In our case, serum IgG was slightly elevated, however it is insufficient to reach the diagnosis. LPRM should be treated surgically, but in this case, the lesion was so diffuse that total resection of tumor was not possible. As for conservative treatment, corticotherapy and azathioprine were prescribed in one case and the tumor size slightly reduced on a 6-month-long follow-up [4]. However, Yang X et al. reported a similar case with poor porgnosis despite the use of corticotherapy [6]. In our case, corticosteroids alleviated symptoms and dural enhancement on post contrast MRI, implying the inflammatory feature of LPRM.

In conclusion, we report a case of LPRM with carpet-like growth pattern, mimicking pachymeningitis. Neuologists should be aware that LPRM can mimic pachymengintis in the early stage. Long-term follow-up is needed for pateints with pachymengiitis and dura matter biopsy are justifiable when the diagnosis of pachymenititis became doubtful.

## Data Availability

Not applicable.

## Abbreviations

MRI: Magnetic resonance imaging
CSF: Cerebrospinal fluid
LPRM: Lymphoplasmacyte-rich meningioma
EPM: *en plaque* Meningiomas
EMA: Epithelial membrane antigen
VIM: Vimentin
PR: Progesterone receptor
IHP: Idiopathic hypertrophic pachymeningitis
